# Candle soot-templated silica nanobiointerface chip for detecting circulating tumour cells from patients with urologic malignancies

**DOI:** 10.1039/c8ra05807e

**Published:** 2018-10-09

**Authors:** Tianying Xing, Binshuai Wang, Yimeng Song, Shudong Zhang, Lulin Ma

**Affiliations:** Department of Urology, Peking University Third Hospital Beijing China malulin@medmail.com.cn

## Abstract

Liquid biopsy, known as fluid biopsy or fluid phase biopsy, is of great clinical significance in cancer diagnosis and treatment monitoring. However, traditional techniques still meet restrictions when aiming for the detection of circulating tumour cells (CTCs) with high efficiency and low cost. Herein, we applied an easily prepared silica nanobiointerface chip for detecting CTCs in prostate cancer (PCa) and clear cell renal cell carcinoma (ccRCC) patients with high efficiency. The silica nanobiointerface chip was fabricated by depositing candle soot on a glass slide, followed by chemical vapour deposition, and then by modifying anti-epithelial cell adhesion molecule (EpCAM) antibody. The silica nanobiointerface chips exhibited excellent abilities to capture PC3 PCa cell lines, with average efficiency of 81.2 ± 1.4%. We demonstrate that the strong topographic interaction between targeted cells and nanostructured surface is critical to enhancing the capture efficiency of CTCs. We further tested peripheral blood samples from 10 preoperative PCa and 7 ccRCC patients. The results show that CTCs from 7 PCa cases and 4 ccRCC cases were successfully detected. We believe that the nanobiointerface chip will provide great potential for the clinical application of CTC.

## Abstract 2

Low-cost and easily prepared efficient nanochips for detecting circulating tumour cells with great clinical potential.

## Graphical abstract

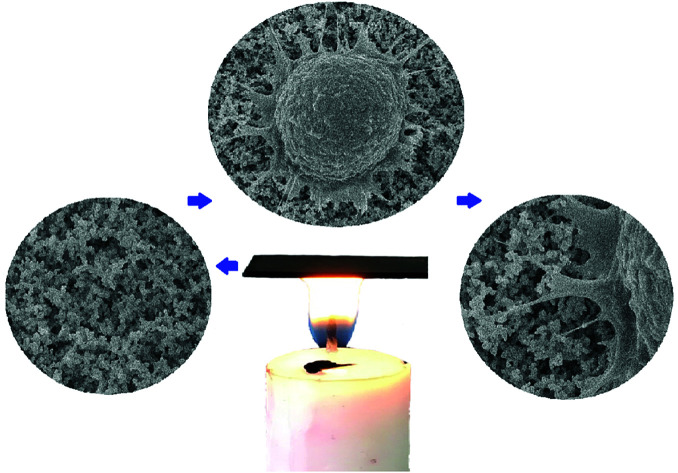

## Introduction

Circulating tumour cells (CTCs) have emerged as a promising biomarker for early diagnosis, monitoring and prognosis of multiple malignancies.^1,2^ Prostate cancer (PCa) is the most common male cancer in western countries and has yielded considerable research on CTCs.^3–5^ However, as another common urologic malignancy, there are limited studies on renal cell carcinoma CTCs. In general, CTC enrichment detection relies on cellular biological properties, such as micro beads, which select CTCs *via* surface marker or deplete unwanted leukocytes,^6,7^ while some assays rely on the physical properties, such as size and density.^8,9^ Despite boosted research interest in CTCs, its low concentrations in peripheral blood poses a serious challenge for any analytical system.^10^ Previous studies on detecting CTCs in PCa patients with the “gold standard” CellSearch System showed controversial correlations between CTC and prognostic factors, resulting from the limited specificity and efficiency of the system.^11–13^ Moreover, the high expense of some detecting assays hampered their clinical application. Developing more efficient methods with low cost is becoming increasingly necessary. In recent years, nanotechnology has shown great promises in CTC enrichment and detection.^14,15^ In addition to the current promising nanosubstrates, we hope to find out more cost-effective and easily-prepared substrates for CTC capturing.

Deng *et al.* have reported a simple way to create nanostructured coating with candle soot.^16^ By holding the glass slide and moving over the stable candle flame, the soot was deposited on the glass substrate; its average density was uniform and height-independent. Chemical vapour deposition with tetraethoxysilane (TEOS) and aqueous ammonia solution was performed, through which a 20 ± 5 nm-thick silica layer was formed surrounding the soot particles. The substrates were then calcined, and carbon cores were thermally degraded, resulting in hollow silica shells. Several nanostructured substrates (such as those with nanopillars) have shown good capability to enrich and capture CTCs from blood due to the enhanced local topographic interactions.^17–19^ Inspired by the irregular dendritic nanostructure, a previous study succeeded in applying the candle soot-templated substrates to capture cell lines and cancer cell-spiked whole mouse blood.^20^ Moreover, its underwater transparency allowed simultaneous monitoring of captured cells, avoiding the interference of unspecific fluorescence. Owing to these promising results and simple fabrication, we detected the CTCs in PCa and ccRCC patients using the candle soot-templated nanobiointerface chips.

## Experimental

### 1. Materials

Glass slides (commercial); white candles (commercial); tetraethyl orthosilicate (TEOS, reagent grade, 98%, Sigma-Aldrich); ammonium hydroxide (AR, 25–28% NH_3_, Beijing Chemical Works); (3-mercaptopropyl)trimethoxysilane (MPTMS, 95%, Sigma-Aldrich); 4-maleimidobutyric acid *N*-hydroxysuccinimide ester (GMBS, ≥98%, Sigma-Aldrich); streptavidin (SA, Invitrogen); ethanol (>99.8%, Beijing Chemical Works); dimethylsulfoxide (DMSO, 99%, Sigma-Aldrich); biotinylated anti-human EpCAM (biotin-anti-EpCAM, R&D systems); biotinylated anti-human carbonic anhydrase 9 (biotin-anti-CA9, R&D systems); biotinylated anti-human CD 147 (biotin-anti-CD147, R&D systems); Dulbecco's modified Eagle medium (DMEM, Invitrogen), phosphate buffer solution (PBS, GE); 4% paraformaldehyde solution (PFA, Solarbio); glutaraldehyde (Ted Pella); hexamethyldisilazane (≥99.9%, Sigma-Aldrich); 4′,6-diamidino-2-phenylindole dihydrochloride (DAPI, Thermofisher Scientific); Triton X-100 (BioXtra, Sigma-Aldrich); bovine serum albumin (BSA, Sigma-Aldrich); CD45 monoclonal antibody, FITC conjugate (Anti-CD45, Thermofisher Scientific); PE-CF594 mouse anti-human cytokeratin (Anti-CK, BD Biosciences).

### 2. Nanobiointerface chip preparation

**Fig. 1:**
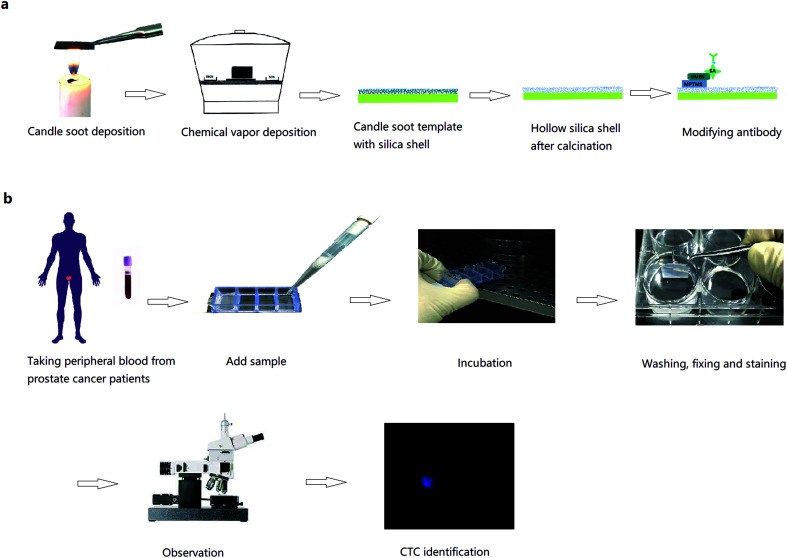
(a) Fabrication of candle soot-templated nanobiointerface chip. (b) Detection of circulating tumour cells in peripheral blood from prostate cancer patients.

Commercial glass slides were cut into 1 × 2 cm pieces to fit Lab-Tek Chamber Slides. The cut slides were held in a candle flame and moved back and forth uniformly for 12 seconds. Then, the slides were placed in a desiccator together with two open glass vessels containing 3 mL TEOS and the other containing ammonium hydroxide. Chemical vapour deposition was performed at 37 °C for 24 hours in an enclosed desiccator. Silica shell was formed by hydrolysis and condensation of TEOS. The substrates were then calcinated at 600 °C for 2 hours in air, and then cooled till room temperature. During calcination, the carbon cores thermally degraded, leaving behind hollow silica shell. After plasma treatment, the substrates were ready for the well-established modification procedures to link biotinylated capturing antibodies (Fig. 1a).^18^ The flat glass substrates were washed and then subjected to plasma treatment and the same modification. To embed the biotin-anti-EpCAM, the substrates were placed in 4-well Lab Tek chamber slides, and then incubated with 25 μL biotin-anti-EpCAM solution (10 μg mL^−1^ in PBS) in each well for 30 min at room temperature. After washed once with PBS, the substrates were ready to use.

### 3. Scanning electron microscope (SEM) images for captured cells

We used diluted PC3 cell suspension (10^5^ cells per mL) for cell capture on nanobiointerface and flat glass chips. After capturing, the chips were washed for 3 times and fixed in 2.5% glutaraldehyde solution at room temperature overnight. Dehydration was then performed using different concentrations of ethanol and purified water (30%, 50%, 70%, 85%, 95% and 100%). Then, the substrates were treated with 50% (in ethanol) and 100% hexamethyldisilazane for 10 min at room temperature, in sequence. After gold sputtering, the chips were observed under SEM (HITACHI, SU8010).

### 4. Cell line capture

PC3 human prostate cancer cell line, Jurkat immortalized human T lymphocyte cell line and Daudi B lymphoblast cell line were cultured according to the general process from American Type Culture Collection (ATCC). For all cell lines, we applied our homemade device (a glass capillary connected to a winged needle set and an injector) to count approximately 50, 100, 200, 500 cells. The counted cells were added in 4-well Lab Tek chamber slides with a biotin-anti-EpCAM-embedded nanobiointerface chip and 1 mL DMEM in each well. PC3 cells were also added to biotin-anti-EpCAM embedded flat glass chips. The cell suspensions in different concentration gradients were all incubated for 45 min (37 °C, 5% CO_2_). Then, the chips were washed with PBS three times, fixed with 4% PFA, washed with PBS and treated by 0.2% Triton X-100. After incubating all the chips in 1% DAPI solution for 10 min, we counted captured cell number using a fluorescence microscope (Leica, TCS SP8). All capturing assays were repeated 3 times for each cell line.

### 5. CTC capture from peripheral blood

All experiments were performed in accordance with the guidelines of “Ethics of Biomedical Research with Human Involved (National Health Commission of P. R. China).” The experiments were approved by the ethics committee of Peking University Third Hospital. Informed consents were obtained from human participants of this study. We randomly chose 10 preoperative prostate cancer patients. Peripheral blood was stored in vacuum tubes with EDTA as an anticoagulant and sent to test as quickly as possible. Then, 1 mL of blood from each patient was added to Lab Tek chamber slides with biotin-anti-EpCAM-embedded chips and incubated for 45 min (37 °C, 5% CO_2_). The chips were then washed gently in PBS 5 times (until there was little gross redness), fixed in 4% PFA for 30 min, washed with PBS for three times and treated with 0.2% Triton X-100 for 30 min. After PBS washing for 3 times and BSA solution (2% BSA in PBS) blocking for 2 hours, the blocking solution was removed without washing. Then, the chips were incubated with 50 μL anti-CK and 50 μL anti-CD45 solution at 4 °C in the dark overnight. After washing with PBS, the chips were incubated in 1% DAPI solution for 5 min and washed again 3 times with PBS. The cells were ready for fluorescence microscopy observation (Leica, TCS SP8, a few by Nikon Ti-E, Fig. 1b). Procedures were the same using blood from 7 renal cancer patients. The chips were incubated with 50 μL mixed antibody solution comprising 25 μL biotin-anti-CA9 (10 μg mL^−1^ in PBS) and 25 μL biotin-anti-CD147 (10 μg mL^−1^ in PBS).

## Results and discussion

**Fig. 2:**
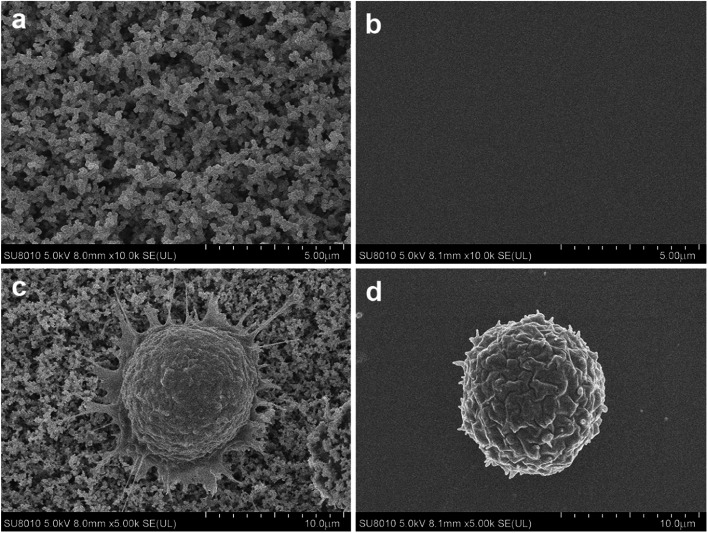
(a) The scanning electron microscope (SEM) image of nanodendritic surface. (b) Flat glass surface without nanostructure. (c) A captured cell with many filopodia protruded on the nanobiointerface chip. (d) A captured cell with regular spherical shape on the flat glass chip.

PCa is the second leading cause of male death according to American Cancer Society, and it is a classic cancer type for CTC research.^3^ First, we applied EpCAM positive PC3 prostate cancer cell suspension for cell capture. Nanobiointerface chips and flat glass chips were put in chamber slides and modified with biotin-anti-EpCAM antibody. PC3 cell suspension was then added to the chamber slides and incubated for 45 min. After cell capturing, we observed the chips *via* SEM. SEM images displayed a nanostructure of irregular dendritic silica shells on the nanobiointerface chip surface (Fig. 2). Previous research showed that cells had limited interactions with the smooth surface of the chip and remained spherical.^17^ In our experiment, however, SEM images revealed that the captured cell morphology changed remarkably with many filopodia protruded on the nanosurface. As a control, cells displayed more regular spherical shape on the flat surface (Fig. 2). These filopodia enhanced the local topographic interactions between cell membrane and nanobiointerface chip surface, which amplified the antigen–antibody reaction and binding affinity.

**Fig. 3:**
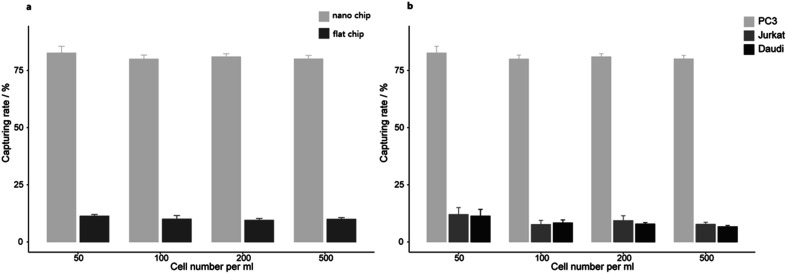
(a) The capturing efficiency of PC3 cells on nanobiointerface chips and flat glass chips. (b) The capturing efficiency of PC3, Jurkat and Daudi cells on nanobiointerface chips.

We further examined the more precise capture efficiency of cell lines. We counted approximately 50, 100, 200 and 500 PC3 cells under the microscope, and also added the cells into 1 mL DMEM in Lab Tek chamber slides with a nanobiointerface or flat glass chips. The cell suspensions in different concentration gradients were all incubated for 45 min (37 °C, 5% CO_2_). After gently washing with PBS for three times, fixing with 4% PFA and staining with DAPI, we counted the captured cells under the fluorescence microscope. On the nanobiointerface chips, the numbers of captured PC3 cells were 41 (41/50, 82%), 77 (77/100, 77%), 167 (167/200, 83.5%), and 403 (403/500, 80.6%), while on flat glass chips, the numbers of captured cells were only 5 (5/50, 10%), 8 (8/100, 8%), 22 (22/200, 11%), and 43 (43/500, 8.6%). All capturing procedures were repeated another two times. Collectively, our nanobiointerface chips showed good capture efficiency with an average of 81.2 ± 1.4%, which is significantly higher than the efficiency of flat chips (Fig. 3). To determine the structure-derived non-specific binding, we also captured EpCAM negative Jurkat and Daudi cells. Under the same four concentration gradients, the average capture efficiency was only 9.2 ± 2.0% and 8.4 ± 2.1% for Jurkat and Daudi cells, respectively (Fig. 3). Compared with the significant lower capture rate of flat chips or EpCAM negative cells, the nanobiointerface chips could enhance the capture ability of targeted cells *via* dual structural and molecular recognition. Although manual errors could occur in the cell adding and counting process, the capture efficiency of the nanobiointerface chips for PC3 cells was comparable to that reported in previous research using such chips and higher than that of traditional SiNP substrates.^17,20^ In addition, we chose the countable cell concentrations analogous to general CTC concentration in cancer patients' blood. The capture efficiency almost remained stable for cell concentration no more than 500/mL.

**Fig. 4:**
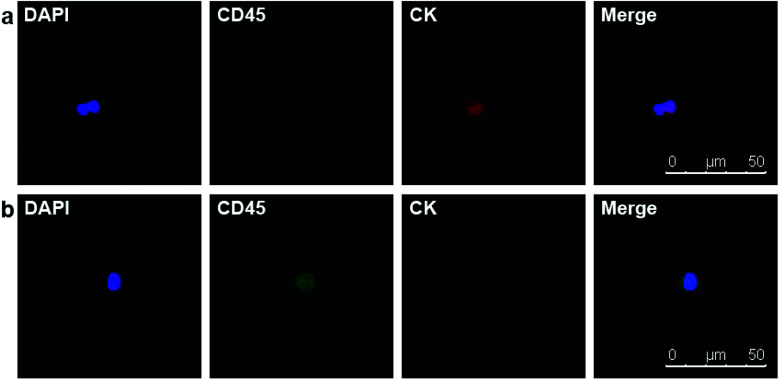
(a) DAPI+/CD45−/CK + CTC in peripheral blood of a PCa patient. (b) DAPI+/CD45+/CK− white blood cell.

Due to the satisfying capture efficiency on cell lines, we further investigated the ability of our chips to catch CTCs under real clinical condition. Peripheral blood was taken from PCa patients preoperatively and transmitted using a vacuum tube for testing as quickly as possible. Then, 1 mL of the blood sample from each patient was added to chamber slides with biotin-anti-EpCAM-embedded chips and incubated for 45 min. The chips were then washed gently five times in PBS to remove excess red blood cells and fixed with PFA. After blocking and penetration, we marked cytokeratin (CK) with red fluorescence, CD45 with green fluorescence and nuclei with blue fluorescence (DAPI) for fluorescent microscope observation. PCa is epithelium originated, so its CTCs maintain the epithelial marker CK most of the time, while CK does not express in blood cells. We defined the cells meeting all the following criteria as CTCs: (1) positive CK and DAPI staining but negative CD45 staining (CK+/CD45−/DAPI+, white blood cells as CK-/CD45+/DAPI+), (2) cellular diameter between 13 μm to 50 μm, and (3) nuclear-cytoplasmic ratio over 50%. We took blood from 10 preoperative PCa patients. All patients underwent laparoscopic radical prostatectomy; the diagnosis of PCa was confirmed by pathology with stage ranging from T2aN0M0 to T3bN1M0, *i.e.*, both localised and local advanced disease and low to high risk classification (Table 1). Of the 10 patients we examined, we could find at least one CTC in 7 cases meeting all the criteria (Fig. 4). Moreover, we examined 5 patients with benign prostate hyperplasia (malignancy was excluded pathologically) and could not find confirmed CTCs in all these five patients. As we only detected CTCs with epithelial phenotype, CTCs undergoing epithelial mesenchymal transition (EMT) were yet to be identified with more markers. It should be noted that CTC formation is an early event in cancer progression,^21^ and we detected CTCs not only in patients with local advanced disease, but also in early localized tumour without using much blood. Our chips may be sensitive for CTC detection with good specificity in PCa patients with relatively lower tumour burden and earlier stage diseases. Furthermore, the good sensitivity of the biointerface chips may also enable their application in diagnosis of suspicious cases and postoperative disease monitoring.

**Table 1:** Clinical features of 10 prostate cancer patients and numbers of CTC detected^a^

Patient number	Clinical stage^b^	Risk classification^c^	CTC number
1	T3aN0M0	High	2
2	T3aN0M0	High	2
3	T3bN1M0	High	3
4	T2cN0M0	High	0
5	T3bN0M0	High	0
6	T3aN1M0	High	1
7	T3aN0M0	High	1
8	T2aN0M0	Low	0
9	T2bN0M0	Intermediate	1
10	T2cN0M0	High	2

a CTC: Circulating Tumour Cell.

b Tumour Node Metastasis (TNM) classification, International Union Against Cancer, 8^th^ edition.

c 2017 European Association of Urology risk groups for biochemical recurrence.

**Fig. 5:**
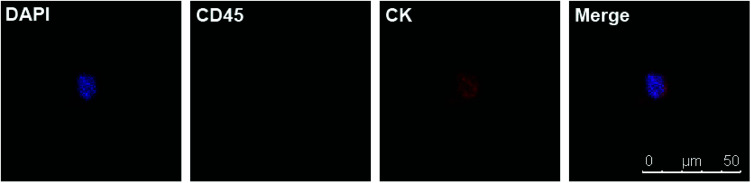
DAPI+/CD45−/CK + CTC in peripheral blood of a ccRCC patient.

**Fig. 6:**
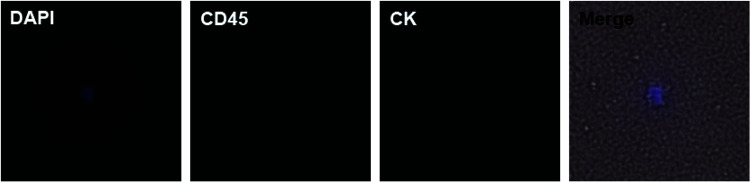
DAPI+/CD45−/CK− suspicious cell (merged with bright field) in peripheral blood of a ccRCC patient. The suspicious cell has larger size than blood cells and irregular shape.

Motivated by the results of PCa, we decided to further detect CTCs in renal cancer patients. Renal cancer (including renal pelvis) is the 5^th^ most common cancer for males and 10^th^ for females.^3^ Unlike prostate cancer, limited studies reported ideal results for detecting CTCs in renal cancer patients.^7,22^ This may result from the insensitivity of both the methods and EpCAM antibody. Clear cell renal cell carcinoma (ccRCC) is the most common type of renal cell carcinoma, but only 10–36.3% of ccRCC cases express EpCAM.^23–25^ One study applied NanoVelcro substrates with anti-carbonic anhydrase 9 (CA9) and CD147 as dual capturing antibodies to test CTCs in 33 ccRCC patients, and 31 of them tested positive. According to this promising result, we further checked the validity of our chips. We chose 7 preoperative ccRCC patients (all confirmed by postoperative pathological assessment) with clinical stage of T3aN0M0 or above and took their peripheral blood (Table 2). The chips were modified with biotin-anti-CA9/CD147 antibodies and blood samples were treated using the same procedures mentioned before. Of the 7 cases, we detected CTCs in 4 cases (57.1%) with the classic phenotype of CK+/CD45−/DAPI+ (Fig. 5). Although the positive rate is lower than the microfluidic NanoVelcro assay, it is comparable or higher than that reported before.^7,22^ Due to the underwater transparency of the chips, we could directly monitor the captured cells *via* bright field imaging. In another case, we found some suspicious cells with the phenotype of CK−/CD45−/DAPI+ and irregular shape but larger size than blood cells (Fig. 6). As previous studies identified CTCs from ccRCC patients acquiring mesenchymal and stem cell phenotypes,^26,27^ we assume that these suspicious cells may be CTCs without epithelial markers. Furthermore, it is possible to identify more phenotypes with our chips and apply other potential capturing markers to enhance positive rate in future. Since we chose the advanced ccRCC cases, the validity of the chips to detect CTCs in earlier stage diseases needs further studies.

**Table 2:** Clinical features of 7 ccRCC patients and numbers of CTC detected

Patient number	Clinical stage^a^	Stage group^b^	CTC number
1	T3aN1M1	IV	3
2	T3aN1M1	IV	2
3	T3aN0M0	III	0
4	T3bN0M1	IV	1
5	T3bN1M0	III	3
6	T3aN0M0	III	0
7	T3aN0M0	III	0 suspicious

a 2017 Tumour Node Metastasis (TNM) classification, International Union Against Cancer, 8^th^ edition.

b Group I, II, III, IV from early to late stage.

## Conclusions

In conclusion, the candle soot-moulded nanobiointerface chips were cost-effective and able to catch specific cancer cells. The irregular dendritic nanostructure enhanced the interaction between cell membrane and chip surface and amplified the binding affinity with targeted cells, contributing to an ideal efficiency of around 80% for PC3 cell line capture. Furthermore, our chips successfully captured CTCs from blood samples of PCa and ccRCC patients. It showed sensitivity to capture CTCs in early-stage PCa patients with low tumour burden and might be valid to distinguish more subtypes of CTCs in both PCa and ccRCC. These low-cost nanobiointerface chips can be used to further study their potential use for early diagnosis of clinically suspicious PCa and postoperative surveillance.

## Conflicts of interest

There are no conflicts to declare.

## Supplementary Material
